# Her Heart's Mistake

**Published:** 1889-08-24

**Authors:** E. D. Cumming

**Affiliations:** Author of "An Ordeal by Silence"


*(To be continued.*

### Her Heart's Mistake.

By E. D. Cumming, Author of "An Ordeal by Silence."

*(Continued from page 316.)*

### Chapter XIX.—"I Dare Not for My Life."

"You tell me that you will not retract your promise without my consent; that you hold yourself bound by it, unless of my own free will I release you. It is well that you have said this; for of my own will I will never let you go. Not three months since you were in my arms, beside yourself with grief that I should leave you. Two weeks ago you wrote to chide me for entertaining what I gladly—ah, Kate, so gladly!—acknowledged to be baseless suspicions. What have I done to forfeit your love? I do not believe that you never cared for me; I cannot believe that you mistook your feelings. You say that you did not know what love was till another man's lips taught you. The lesson has been quickly learned, Kate; so quickly that your pleading loses its power to convince. I love you; you have given me your solemn promise to be my wife, and to that I hold you."

This was the last part of the letter Kate received from George Bairdsley in answer to the one she wrote imploring him to release her from her engagement. He began by loading her with the bitterest reproaches for her inconstancy, but seemed to have grown calmer as he wrote on, and the last pages of his letter pressed far more heavily upon her than the first. She could not bring herself to understand that he refused to let her go. She had thought him a proud man; surely no man having the dimmest spark of pride would insist upon marrying a girl who not only did not love him, but loved another. She had not expected this; her father had brushed aside, without a moment's hesitation, the idea that he would claim an unwilling bride. Dick had told her that any man worthy the name would scorn to do so. She stood in the drawing-room, gazing at the letter, dreamily and sadly, as though its reality were doubtful. Was it possible that her father and lover were wrong after all, and that George Bairdsley claimed only his due? Was she hopelessly committed by that rash, ill-considered promise he had wrung from her almost by force? She need not have given it; she might have let him go. Would God, she had, hard as then it seemed. She had given the promise, and renewed it often, often since unasked, in her kindly pity when he lay ill; and she would not break it now, though to keep it cost her all that made life worth living.

She heard her father's step on the verandah and went out to meet him with the letter in her hand.

"Is that Bairdsley's reply?" asked the Doctor.

Kate gave it to him and bade him read it.

"Tell me what I ought to do, father," she said. "He cannot mean what he says there."

Dr. Harrison sat down on the arm of a chair, took off his helmet, and began to peruse the sheets, his brow growing darker and darker as he read on.

"You are free, Kate," he said, when he had reached the end. "No one will blame you for setting your promise aside after this."

"I will not retract it unless he gives me leave," she said, firmly. "I *must* abide by it if he insists."

"You are wrong, dear," answered the Doctor; "but I will write to Bairdsley myself if you want his consent to clear your conscience. It's absurd to suppose that he wishes to marry a woman who doesn't care for him. He must know that he is spinning a cord to hang himself. Would you like me to write to him?"

Kate reflected for a few moments. She would have preferred to have conducted such a correspondence as this entirely by herself, but she could not refuse such aid at such a crisis, and asked him to communicate with Bairdsley at once. She, also, would write to him again, and appeal to his generosity to set her free.

Dr. Harrison undertook to write that evening, but at the same time he warned her that he could not do so for the express purpose of enabling her to marry Mr. Warren. He would simply tell Bairdsley that he had satisfied himself that his daughter could not give him the affection he had a right to expect from the girl who married him; and he had no doubt whatever that very little consideration would induce the man to absolve her from her promise. He could understand how fiercely he must have resented being asked to resign his claim to her hand in favour of another. Kate had been right in telling him the whole truth, but it would serve no good purpose to dwell upon her love for Warren. And, moreover, he was bound to keep a free hand to deal with the barrister's aspirations afterwards.

And Kate listened, and agreed. She was willing that her father should do what he considered best: she would not think about Warren now; she held herself tied by the promise she had so unwisely given, and until Bairdsley released her from it she would continue to regard herself as engaged to him.

So two letters to Simla went on their way that evening, and Kate watched and waited in burning anxiety for the replies. They came by return of post, and she tore open that addressed to herself, prepared for a fierce outburst of jealous anger and a scornful bidding to do what she would. The answer stunned her when she read it.

"You are mine and mine only," wrote Bairdsley. "I care nothing for the love you have given to another man. You have pledged your word to marry me and I hold you to it. You loved me dearly for a few months; you will love him for as long, unless some other suitor comes to banish him. You say you like me still as you always have done. I thank you, Kate; it is enough for me, and I will marry you, firm in the belief that your liking will rekindle to its old warmth. After what you have told me about Mr. Warren's regard for you and your affection for him, I need hardly point out that I cannot allow the girl who is engaged to me to maintain such an intimacy. Mr. Warren will, I am sure, be of my opinion, that his best and proper course is to leave Meerut at once."

Kate dropped the letter and sank down. How could she convince him that she had never loved him? Had she unconsciously acted a part so well? And when she came to think of her past devotion to him, her endless letters, she could not wonder that he was loth to grant her release. He had long since fallen from the hero's pedestal he once occupied, but she had never allowed him to know it. It was hard to make him realise that all those caresses and endearments had been received and given by mistake. By mistake! How painfully grotesque it was! Bairdsley's letter to her father was brief and formal. In it the writer expressed his surprise that the Doctor should ask him to liberate his daughter from her engagement. What, he asked, would have been thought of him if *he* had discovered that he had misconstrued his feelings, and begged to be freed from his promise to marry her? He could not entertain the request that had been made, and felt sure that he claimed no more than his due in expecting Kate to keep faith with him.

The Doctor bit his lip with anger when he read the letter.

"That I should ever have allowed the cur to cross my threshold!" he muttered under his breath, "that I should ever have given my sanction to his marrying Kate. This is the polished, gentlemanly Captain Bairdsley! Heaven help us, what living lies this world contains!"

Richard Warren came in that evening, and the Doctor told him of the letters which had been received.

"I would rather see her lying in her coffin than give her to such a fellow as this," he said, "but I cannot persuade her that she has done everything she can do to repair her error, and has a right to consider herself free. Try what you can do, Warren; you will find her in the little room beside the drawing-room."

Warren went to the door and raising the purdah, asked if he might come in. Kate was seated before the writing-table resting her head upon her hand, trifling with the letter that afternoon's post had brought her.

"He will not let me go, Dick," she said in a tone of resigned misery. "There's his letter. Read it."

Warren took it, and Kate sat watching his face. He looked sterner than she had ever seen him look before as he turned over one sheet after another.

"Captain Bairdsley is kind enough to suggest that I should go away," he said, laying down the letter.

Kate drew a deep breath and turned to him, still resting her elbow upon the table.

"You must go, Dick; it will be better for us both. I cannot go back upon my word, and since he insists upon it I am bound to marry him."

"I will go to-morrow on one condition," said Warren, ignoring the latter part of her speech.

"What is it?"

"That you come with me."

She smiled faintly and shook her head.

"Will you not go for my sake, Dick?" she asked.

"Kate, this is the purest folly. You have done more, far more, than any girl need do to atone for a rash promise, and still you persist in considering yourself engaged to the man. I cannot understand why you hug your mistake thus when it is eating your very heart away. Throw it from you boldly, and tell Bairdsley that you have done so. Tell him that you will hear nothing more from him, and that you have written your last word. Will nothing I can say convince you that you are making a second mistake worse than the first? Can you not see that this man's meanness seeks shelter behind your high sense of honour, and that he will never yield in his cowardly selfishness? He only seeks to gratify his own passion, and cares nothing for your happiness. Does such a nature deserve the treatment you bestow upon it?"

"Were he ten times worse, Dick, I could not break my word."

Warren remained arguing and reasoning with her for nearly an hour, and then, in despair, returned to seek her father.

"She is absolutely immovable," he said. "She has got an idea that this promise of hers is inviolably sacred, and nothing will persuade her that she is wrong."

"I trust she will think better of it before long. Captain Bairdsley has shown me a phase of his character which has quite wrested my sympathies from him."

"I cannot understand how any man could so far demean himself as to take advantage of a girl's mistaken sense of honour."

"I always thought him a selfish man," said the Doctor; "but I confess that the arguments he put forward in the note he wrote to me opened my eyes."

Warren went back to Kate's room to renew his entreaties. He found her in the same attitude, staring with dry, unseeing eyes at the letter on the table; there was a grey, drawn look upon her face which spoke eloquently of the mental agony she endured, and Warren clenched his teeth with impotent rage as he sat down beside her.

"Are you going away, Dick?" she asked.

"No, certainly not; unless you will marry me and come, too. Why will you not see the terrible life you are allowing yourself to be drawn into, Kate? If you dread meeting this man, I will marry you at once and we'll leave the station. I have a little money, and my profession, and we shall not starve. Can you not trust yourself to me?"

"I would go anywhere with you."

"Then why not trust your conscience to me also? Why not let me think for you in this? I would not counsel you to do a dishonourable act, and I know you would not listen to me if I did."

"Oh, Dick, don't try to persuade me."

There was little in the words; but the hopeless dejection and misery in her expression and manner drove Warren almost wild with love and hate. He could not trust himself to speak, and went away trembling with confused emotions. It would have gone hard with Bairdsley had his rival met him that night.

After he had gone, Kate once more roused herself to write to her affianced husband. She had fallen into a species of dull stupor, and was half unconscious of her surroundings. But now she braced her energies, and made one more effort to obtain her freedom.

"I have done you a bitter wrong," she wrote, "and I cannot repair it, for even if you hold me to my word I shall be your wife only in name. George, am I worth hunting down thus? Have you so little pride that you can let me fetter myself with a promise? If you are not dead to all sense of common humanity, I ask you once more to release me. Even the liking I once had for you is fading into hate before your selfishness."

Three days dragged by, but Bairdsley did not answer. "He means to let you go," said the Doctor. "If he doesn't I shall simply forbid him the house when he returns."

His anxiously looked-for reply came at last, four days later than it might had Kate's appeal been answered at once. It was curt and angry. He told her in as many words that he would marry her if she hated him.

